# Meal Frequencies Modify the Effect of Common Genetic Variants on Body Mass Index in Adolescents of the Northern Finland Birth Cohort 1986

**DOI:** 10.1371/journal.pone.0073802

**Published:** 2013-09-10

**Authors:** Anne Jääskeläinen, Ursula Schwab, Marjukka Kolehmainen, Marika Kaakinen, Markku J. Savolainen, Philippe Froguel, Stéphane Cauchi, Marjo-Riitta Järvelin, Jaana Laitinen

**Affiliations:** 1 Department of Clinical Nutrition, Institute of Public Health and Clinical Nutrition, University of Eastern Finland, Kuopio, Finland; 2 Institute of Clinical Medicine, Internal Medicine, Kuopio University Hospital, Kuopio, Finland; 3 Biocenter Oulu, University of Oulu, Oulu, Finland; 4 Institute of Health Sciences, University of Oulu, Oulu, Finland; 5 Institute of Clinical Medicine, University of Oulu, Oulu, Finland; 6 Clinical Research Center, Department of Internal Medicine, Oulu University Hospital, Oulu, Finland; 7 Department of Genomics of Common Disease, School of Public Health, Imperial College London, London, United Kingdom; 8 CNRS UMR 8199, Lille Pasteur Institute, Lille, France; 9 Lille II University, Lille, France; 10 European Genomic Institute for Diabetes (EGID), Lille, France; 11 Department of Children and Young People and Families, National Institute for Health and Welfare, Oulu, Finland; 12 Department of Epidemiology and Biostatistics, MRC-HPA Centre for Environment and Health, School of Public Health, Imperial College London, London, United Kingdom; 13 Unit of Primary Care, Oulu University Hospital, Oulu, Finland; 14 Finnish Institute of Occupational Health, Oulu, Finland; Tulane School of Public Health and Tropical Medicine, United States of America

## Abstract

Recent studies suggest that meal frequencies influence the risk of obesity in children and adolescents. It has also been shown that multiple genetic loci predispose to obesity already in youth. However, it is unknown whether meal frequencies could modulate the association between single nucleotide polymorphisms (SNPs) and the risk of obesity. We examined the effect of two meal patterns on weekdays –5 meals including breakfast (regular) and ≤4 meals with or without breakfast (meal skipping) – on the genetic susceptibility to increased body mass index (BMI) in Finnish adolescents. Eight variants representing 8 early-life obesity-susceptibility loci, including *FTO* and *MC4R*, were genotyped in 2215 boys and 2449 girls aged 16 years from the population-based Northern Finland Birth Cohort 1986. A genetic risk score (GRS) was calculated for each individual by summing the number of BMI-increasing alleles across the 8 loci. Weight and height were measured and dietary data were collected using self-administered questionnaires. Among meal skippers, the difference in BMI between high-GRS and low-GRS (<8 and ≥8 BMI-increasing alleles) groups was 0.90 (95% CI 0.63,1.17) kg/m^2^, whereas in regular eaters, this difference was 0.32 (95% CI 0.06,0.57) kg/m^2^ (*p*_interaction_  = 0.003). The effect of each *MC4R* rs17782313 risk allele on BMI in meal skippers (0.47 [95% CI 0.22,0.73] kg/m^2^) was nearly three-fold compared with regular eaters (0.18 [95% CI -0.06,0.41] kg/m^2^) (*p*_interaction_  = 0.016). Further, the per-allele effect of the *FTO* rs1421085 was 0.24 (95% CI 0.05,0.42) kg/m^2^ in regular eaters and 0.46 (95% CI 0.27,0.66) kg/m^2^ in meal skippers but the interaction between *FTO* genotype and meal frequencies on BMI was significant only in boys (*p*_interaction_  = 0.015). In summary, the regular five-meal pattern attenuated the increasing effect of common SNPs on BMI in adolescents. Considering the epidemic of obesity in youth, the promotion of regular eating may have substantial public health implications.

## Introduction

Prevention of obesity in children has been proposed as a public health priority to combat the obesity epidemic. However, the etiology of childhood obesity is multifaceted with both lifestyle and genetic factors playing a role in the susceptibility to excessive weight gain [1].

In recent years, numerous obesity-related genetic loci have been identified through genome-wide association studies (GWAS) [2]. Although most of the GWAS for obesity have focused on adult body mass index (BMI), several adult-discovered genetic determinants contribute to common childhood obesity as well [3]–[6]. Among the well-established genetic factors that influence weight development already in childhood are the common single nucleotide polymorphisms (SNPs) in the *FTO* and *MC4R* gene regions [7]–[10].

The dietary causes of obesity are complex and incompletely understood. In relation to childhood and adolescent obesity, it has been proposed that the impact of overall eating patterns may be more significant than that of single foods or nutrients [11]. Some studies indicate that higher meal frequencies and regular breakfast consumption are inversely associated with obesity in youth [12], [13], while some studies have failed to detect any association [14], [15].

Studies on interaction between genetic and lifestyle factors on obesity are emerging, although to a lesser extent in young populations than in adults. In Spanish children, dietary fat composition was found to modify the association between the *FTO* gene variant rs9939609 and obesity risk [16]. A recent meta-analysis including both adults and children concluded that physical activity attenuated the effect of *FTO* variants on obesity risk in adults but not in children [17]. However, our previous study on variation in the *FTO* gene in the Northern Finland Birth Cohort 1986 (NFBC1986) indicated that in highly physically active adolescents the risk of higher BMI was significantly attenuated even among those carrying two risk alleles; nearly down to the level of those having no risk alleles [10]. These results suggest that the effect of common obesity-susceptibility gene variants could be modified by environmental factors.

Previously, we reported an association of a regular five-meal-a-day pattern with reduced risks of overweight/obesity and abdominal obesity in youth [18]. In the present study, we evaluate the impact of two meal frequencies, i.e., 5 meals a day and ≤4 meals a day, on the association between obesity-related genotypes and body mass index among 16-year-old Finnish adolescents. The results indicate that regular meal frequency attenuates genetic predisposition to increased BMI in terms of both single gene variants (*FTO* rs1421085 and *MC4R* rs17782313) and a multiple-locus indicator (a genetic risk score based on eight obesity-susceptibility loci).

## Materials and Methods

### Ethics statement

The study was approved by the Ethics Committee of the Faculty of Medicine of the University of Oulu and written informed consent was obtained from both adolescents and their parents according to the Declaration of Helsinki.

### Study population

The Northern Finland Birth Cohort 1986 (NFBC1986) is an ongoing, population-based study which at the baseline comprised 9432 infants born alive in the two northernmost provinces of Finland to women with expected delivery dates between July 1, 1985 and June 30, 1986, covering 99% of all eligible births in the region. Data have been collected prospectively since the prenatal period, as previously described [19]. At the 16-year follow-up in 2001–2002, 80% (n = 7344) of the adolescents responded to a postal questionnaire concerning their health behavior and well-being and 74% (n = 6798) participated in a clinical examination. The current analysis included individuals for whom data on height, weight, stage of puberty, meal frequency on weekdays at 16 years, and all chosen BMI-related SNPs were available (2215 boys and 2449 girls).

### Clinical examination

In 2001–2002, a clinical examination on adolescents was carried out in municipalities of Northern Finland and also in major cities elsewhere in Finland. The trained study personnel (three teams each consisting of one laboratory analyst and two study nurses) performed the examinations according to a standardized protocol. Venous blood samples were drawn for DNA extraction and anthropometric measurements (height in centimetres, weight in kilograms to one decimal place) were performed. BMI was calculated as weight in kilograms divided by height in meters squared. At the clinical examination, the adolescents self-assessed their pubertal maturation using gender-specific line drawings of the Tanner puberty stages [20].

### Meal patterns

At the 16-year follow-up, adolescents filled in a postal questionnaire on their health behavior and well-being including a question about meal consumption on weekdays. The frequency of meals was assessed with a question ‘Do you usually have the following meals (breakfast, lunch, snack, dinner, and evening snack) on weekdays?’ with dichotomous response options (yes/no). For the analyses, adolescents were classified into two groups by their meal consumption: five meals a day including breakfast (regular meal pattern) or ≤ four meals a day (meal skipping), the latter including both regular breakfast eaters and breakfast skippers.

### Genotyping

We genotyped eight SNPs, rs1421085, rs17782313, rs6265, rs10938397, rs1424233, rs6548238, rs11084753, and rs2815752, representing the childhood obesity-susceptibility loci, identified also in recent GWAS [2]–[6], at or near the *FTO* (fat mass- and obesity-associated), *MC4R* (melanocortin 4 receptor), *BDNF* (brain-derived neurotrophic factor), *GNPDA2* (glucosamine-6-phosphate deaminase 2), *MAF* (v-maf musculoaponeurotic fibrosarcoma oncogene homolog), *TMEM18* (transmembrane protein 18), *KCTD15 (*potassium channel tetramerization domain containing 15), and *NEGR1* (neuronal growth regulator 1) genes, respectively. The DNA extractions, sample quality controls, biobank up-keeping and aliquoting were performed in the National Public Health Institute, Biomedicum Helsinki, Finland. Genotyping was performed using TaqMan single nucleotide polymorphism assay (Applied Biosystems, Foster City, CA). The Ensembl gene IDs for the reported genes are: *FTO*: ENSG00000140718; *MC4R*: ENSG00000166603; *BDNF*: ENSG00000176697; *GNPDA2*: ENSG00000163281; *MAF*: ENSG00000178573; *TMEM18*: ENSG00000151353; *KCTD15*: ENSG00000153885; *NEGR1*: ENSG00000172260.

### Statistical analyses

For the genetic risk score (GRS), we used the above eight SNPs representing BMI-associated loci. SNP genotypes were recoded as 0, 1, or 2 BMI-increasing alleles and the risk score was created by adding up the number of these alleles. Due to lack of established effect sizes for each of the SNPs and since it is unadvisable to generate weights from the data under analysis [21], the risk alleles were not weighted by their effect size. For the analysis of interaction between genetic susceptibility and meal frequencies, the sample was divided into high-risk and low-risk groups using the median value of the GRS (8) as the cut- off point. For the *FTO* and *MC4R* variants, the interaction effects of the genotype and meal frequencies on BMI were also analyzed separately using an additive model of inheritance. BMI was treated as a continuous variable and gene-diet interactions were investigated using linear regression analysis (GRS as a continuous variable) and analysis of variance (ANOVA; genotypes as categorical variables) adjusted for gender and Tanner stage of puberty (numbered 1–5). Hardy-Weinberg equilibrium and associations between genotypes and meal frequencies were tested using the chi-squared test. In order to avoid excessive testing for statistical significance, the results are reported as mean values with either their 95% confidence intervals (CI) which demonstrate reliability of the estimates or their standard deviations (SD). The 19 *p*-values related to testing of pre-set hypotheses in this paper have not been corrected for multiple testing. The statistical analyses were performed using SPSS Statistics, version 17.0 (SPSS Inc., Chicago, IL, USA).

## Results

All variants passed the quality control criteria (call rate >95% and in Hardy-Weinberg equilibrium [*p*>0.05]) and had minor allele frequency ≥0.16 (Table 1). The mean BMI in the total sample was 21.2 kg/m^2^ (SD 3.4) and the mean age of adolescents was 16.0 (SD 0.4) years. Table 2 shows mean values of BMI across genotype, meal pattern and puberty stage categories. For the individuals with GRS ≥8 (high-risk group), the mean BMI was 0.7 units greater (21.5 [95% CI 21.3, 21.6] kg/m^2^) than that for those with GRS <8 (low-risk group) (20.8 [95% CI 20.7, 21.0] kg/m^2^). Carriers of two risk alleles in *FTO* rs1421085 had an increased BMI (21.7 [95% CI 21.5, 22.0] kg/m^2^) compared with individuals with 0 or 1 risk allele (20.9 [95% CI 20.8, 21.1] kg/m^2^ and 21.2 [95% CI 21.0, 21.3] kg/m^2^, respectively). Similarly, carrying both of the risk-conferring alleles of rs17782313 at the *MC4R* locus was associated with a greater BMI (22.2 [95% CI 21.6, 22.9] kg/m^2^) compared with the other two genotypes (TT: 21.1 [95% CI 21.0, 21.2] kg/m^2^ and CT: 21.3 [95% CI 21.1, 21.5] kg/m^2^).

**Table 1:** Genotype information and quality control statistics for the eight childhood obesity-susceptibility SNPs.

SNP	Chromosome	Nearest gene	Position (bp)	Risk allele	Non-risk allele	MAF	HWE *p*-value	Original reported lead SNP	Reference
rs1421085	16	*FTO*	53,800,954	C	T	0.41	0.59	rs9939609	[3], [6]
rs17782313	18	*MC4R*	57,851,097	C	T	0.18	0.72	rs17782313	[3], [6]
rs6265	11	*BDNF*	27,679,916	T	C	0.16	0.49	rs925946	[3]
rs10938397	4	*GNPDA2*	45,182,527	G	A	0.49	0.68	rs10938397	[3]
rs1424233	16	*MAF*	79,682,751	T	C	0.43	0.94	rs1424233	[6]
rs6548238	2	*TMEM18*	634,905	C	T	0.16	0.98	rs6548238	[3]
rs11084753	19	*KCTD15*	34,322,137	G	A	0.34	0.64	rs11084753	[3]
rs2815752	1	*NEGR1*	72,812,440	A	G	0.36	0.63	rs2815752	[3]

Abbreviations: bp, base pairs; HWE, Hardy-Weinberg equilibrium; MAF, minor allele frequency; SNP, single nucleotide polymorphism.

**Table 2:** Distributions of eight single nucleotide polymorphisms, genetic predisposition score, Tanner stages of puberty, meal patterns and body mass index among adolescents in the Northern Finland Birth Cohort 1986.

	Boys (n = 2215)		Girls (n = 2449)		All (n = 4664)	
	Mean BMI kg/m^2^ (95% CI)	%	Mean BMI kg/m^2^ (95% CI)	%	Mean BMI kg/m^2^ (95% CI)	%
*FTO* rs1421085						
TT	20.8 (20.5, 21.0)	35.9	21.1 (20.8, 21.3)	34.9	20.9 (20.8, 21.1)	35.4
CT	21.1 (20.9, 21.3)	47.9	21.2 (21.0, 21.4)	48.2	21.2 (21.0, 21.3)	48.0
CC	21.8 (21.4, 22.2)	16.2	21.7 (21.3, 22.0)	16.9	21.7 (21.5, 22.0)	16.6
*MC4R* rs17782313						
TT	21.1 (20.9, 21.2)	69.1	21.1 (20.9, 21.2)	67.5	21.1 (21.0, 21.2)	68.2
CT	21.1 (20.8, 21.4)	27.8	21.5 (21.2, 21.7)	29.2	21.3 (21.1, 21.5)	28.5
CC	21.9 (21.1, 22.7)	3.1	22.5 (21.6, 23.5)	3.3	22.2 (21.6, 22.9)	3.2
*BDNF* rs6265						
CC	21.0 (20.9, 21.2)	70.1	21.2 (21.1, 21.4)	70.3	21.1 (21.0, 21.2)	70.2
CT	21.3 (21.0, 21.5)	27.2	21.2 (21.0, 21.5)	27.2	21.2 (21.1, 21.4)	27.2
TT	21.4 (20.5, 22.4)	2.7	21.3 (20.4, 22.2)	2.5	21.4 (20.7, 22.0)	2.6
*GNPDA2* rs10938397						
AA	21.1 (20.9, 21.4)	26.4	20.9 (20.7, 21.1)	26.9	21.0 (20.9, 21.2)	26.7
AG	21.0 (20.8, 21.2)	50.0	21.3 (21.1, 21.5)	48.5	21.2 (21.0, 21.3)	49.2
GG	21.2 (20.9, 21.6)	23.6	21.4 (21.1, 21.7)	24.6	21.3 (21.1, 21.5)	24.1
*MAF* rs1424233						
CC	21.1 (20.9, 21.4)	32.3	21.0 (20.8, 21.2)	32.0	21.1 (20.9, 21.2)	32.2
CT	21.0 (20.7, 21.2)	48.7	21.3 (21.1, 21.5)	50.4	21.1 (21.0, 21.3)	49.6
TT	21.5 (21.1, 21.8)	19.0	21.4 (21.1, 21.8)	17.6	21.5 (21.2, 21.7)	18.2
*TMEM18* rs6548238						
TT	20.2 (19.3, 21.2)	2.1	20.5 (20.0, 21.1)	2.5	20.4 (19.9, 20.9)	2.3
CT	21.1 (20.8, 21.3)	27.1	21.2 (20.9, 21.4)	25.8	21.1 (20.9, 21.3)	26.4
CC	21.2 (21.0, 21.3)	70.8	21.3 (21.1, 21.4)	71.7	21.2 (21.1, 21.3)	71.2
*KCTD15* rs11084753						
AA	21.0 (20.5, 21.4)	12.2	21.1 (20.8, 21.5)	11.2	21.1 (20.8, 21.4)	11.7
AG	21.1 (20.9, 21.3)	45.6	21.2 (21.0, 21.4)	45.5	21.1 (21.0, 21.3)	45.5
GG	21.1 (20.9, 21.4)	42.2	21.3 (21.1, 21.5)	43.3	21.2 (21.1, 21.4)	42.8
*NEGR1* rs2815752						
GG	20.8 (20.4, 21.1)	13.8	20.9 (20.6, 21.3)	12.3	20.8 (20.6, 21.1)	13.0
AG	20.9 (20.7, 21.1)	46.1	21.2 (21.0, 21.4)	47.0	21.1 (20.9, 21.2)	46.6
AA	21.5 (21.2, 21.7)	40.1	21.4 (21.2, 21.6)	40.7	21.4 (21.2, 21.6)	40.4
Genetic risk score <8	20.8 (20.6, 21.0)	48.7	20.9 (20.7, 21.0)	47.3	20.8 (20.7, 21.0)	48.0
Genetic risk score ≥8	21.4 (21.2, 21.6)	51.3	21.5 (21.4, 21.7)	52.7	21.5 (21.3, 21.6)	52.0
Tanner stage of puberty						
II	17.9 (15.7, 20.1)	0.5	20.7 (18.3, 23.1)	0.3	18.9 (17.3, 20.6)	0.4
III	20.6 (20.0, 21.2)	6.8	20.3 (20.0, 20.6)	15.5	20.4 (20.1, 20.7)	11.3
IV	20.6 (20.4, 20.9)	38.1	21.1 (20.9, 21.2)	57.1	20.9 (20.8, 21.0)	48.1
V	21.5 (21.3, 21.7)	54.6	22.1 (21.8, 22.4)	27.1	21.7 (21.6, 21.9)	40.2
Meal pattern						
Regular (five meals a day with breakfast)	20.6 (20.4, 20.8)	53.6	20.8 (20.6, 21.0)	40.5	20.7 (20.6, 20.8)	46.7
Meal skipping (≤4 meals a day)	21.7 (21.4, 21.9)	46.4	21.5 (21.4, 21.7)	59.5	21.6 (21.5, 21.7)	53.3

Data presented as mean values (95% CIs) and percentages.

Abbreviations: BMI, body mass index; CI, confidence interval.

Initially, meal frequency variable consisted of three categories, i.e., five meals including breakfast, ≤ four meals including breakfast (semi-regular meal pattern) and ≤ four meals not including breakfast (breakfast skipping) [18]. As there was no difference in the mean BMI between the latter two groups (21.5 (95% CI 21.3, 21.7) kg/m^2^ and 21.7 (95% CI 21.5, 22.0) kg/m^2^, respectively) they were combined into one category characterized by meal skipping. Regular eaters had lower BMI (20.7 [95% CI 20.6, 20.8] kg/m^2^) than meal skippers (21.6 [95% CI 21.5, 21.7] kg/m^2^) (Table 2). There was no association between the two meal patterns and GRS categories (*p* = 0.292), *FTO* rs1421085 (*p* = 0.855) or *MC4R* rs17782313 (*p* = 0.596).

**Figure 1:**
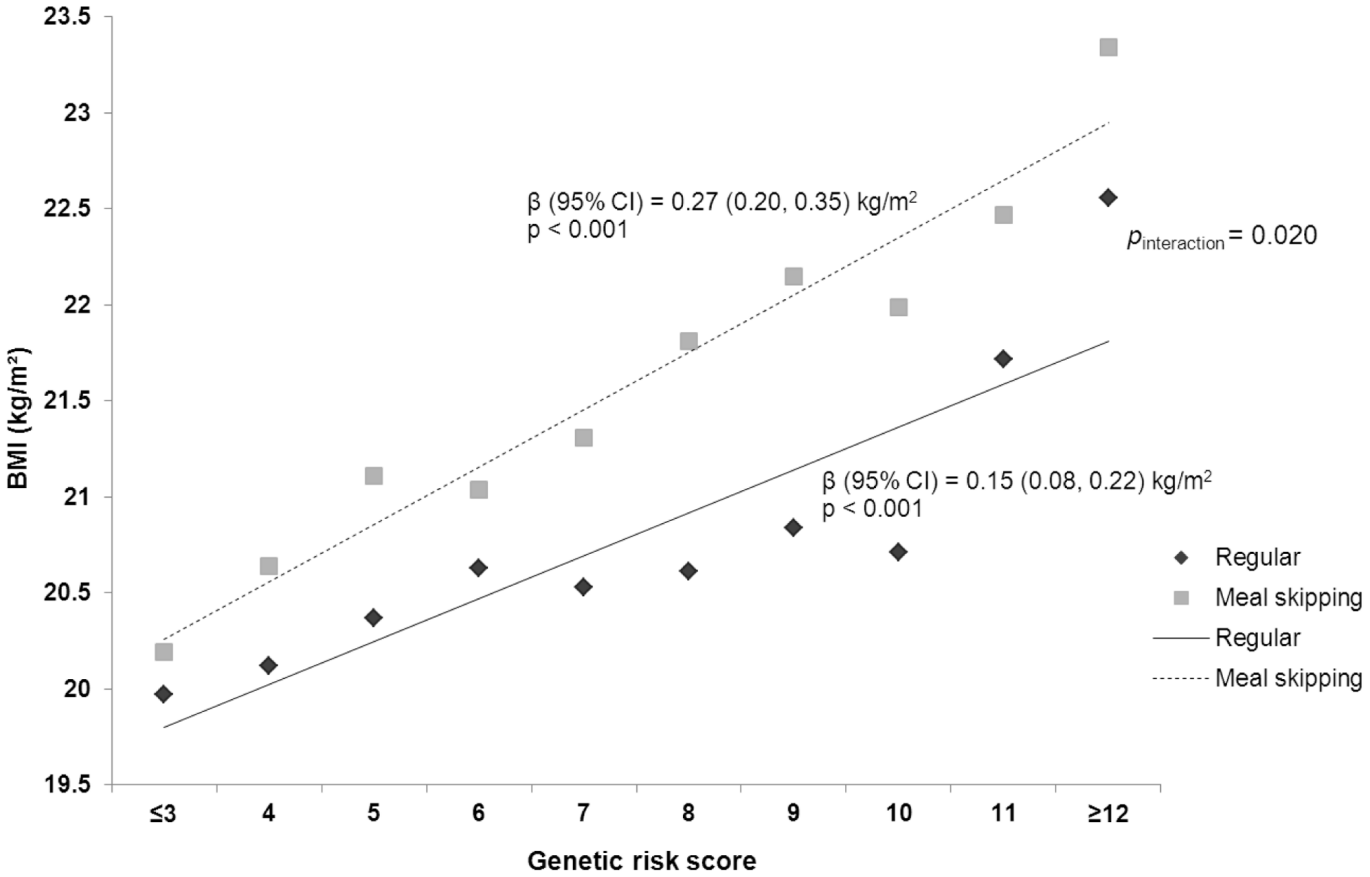
Cumulative effect of genetic risk score (per allele effect) on BMI by meal patterns.

Among the whole population, in the linear regression analysis adjusted for gender and pubertal stage, each additional BMI-increasing allele in the GRS was associated with a 0.21 (95% CI 0.16, 0.26) kg/m^2^ increase in BMI. We then looked at the effects of GRS on BMI separately for the two meal patterns and found that among meal skippers the increase in BMI was 0.27 (95% CI 0.20, 0.35) kg/m^2^ per risk allele, whereas among regular eaters this effect was only 0.15 (95% CI 0.08, 0.22) kg/m^2^ (*p*_interaction_  = 0.020; Figure 1).

**Figure 2:**
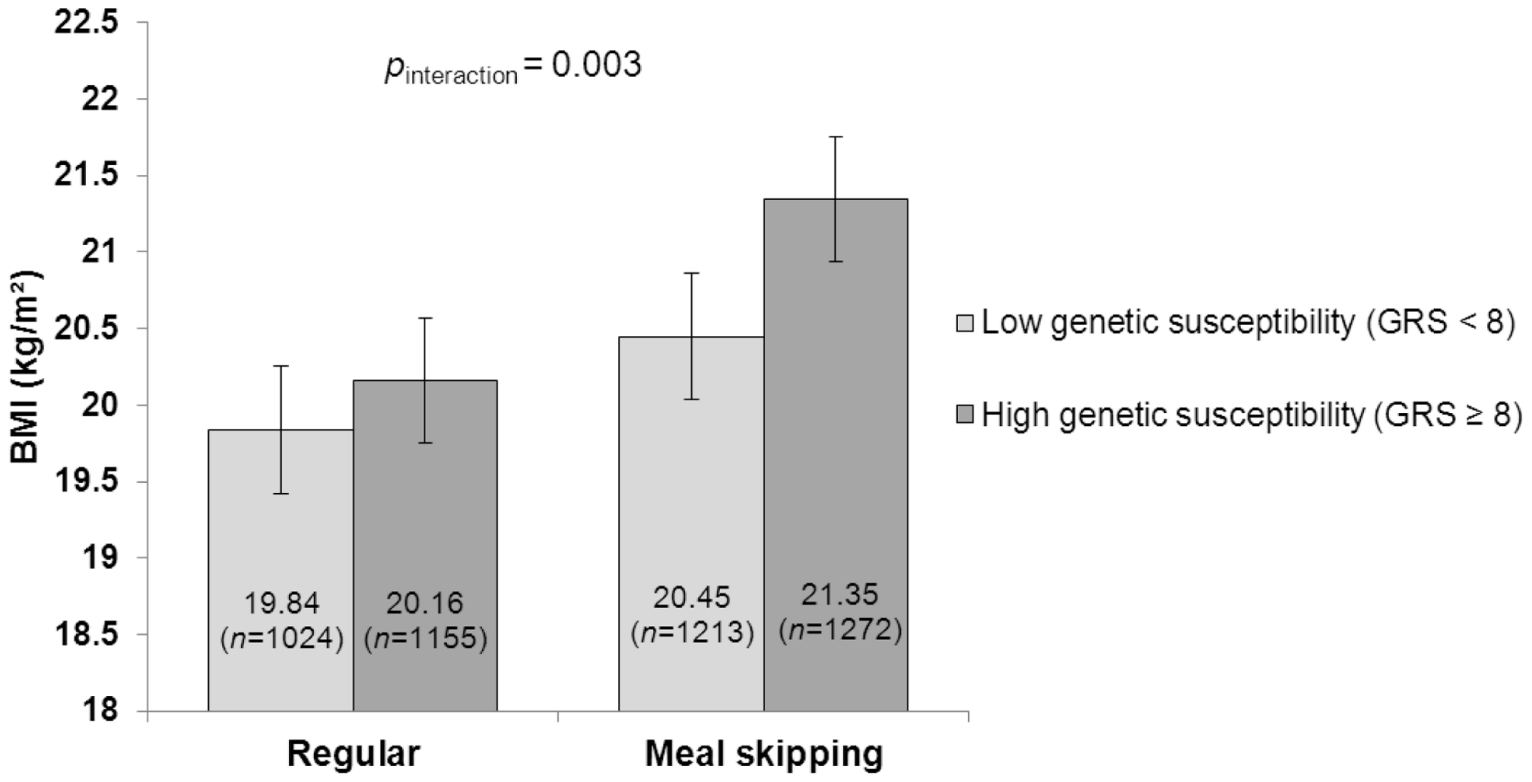
Interaction between genetic risk score (GRS) and meal patterns on BMI. Mean BMI values with 95% confidence interval error bars are presented.

**Figure 3:**
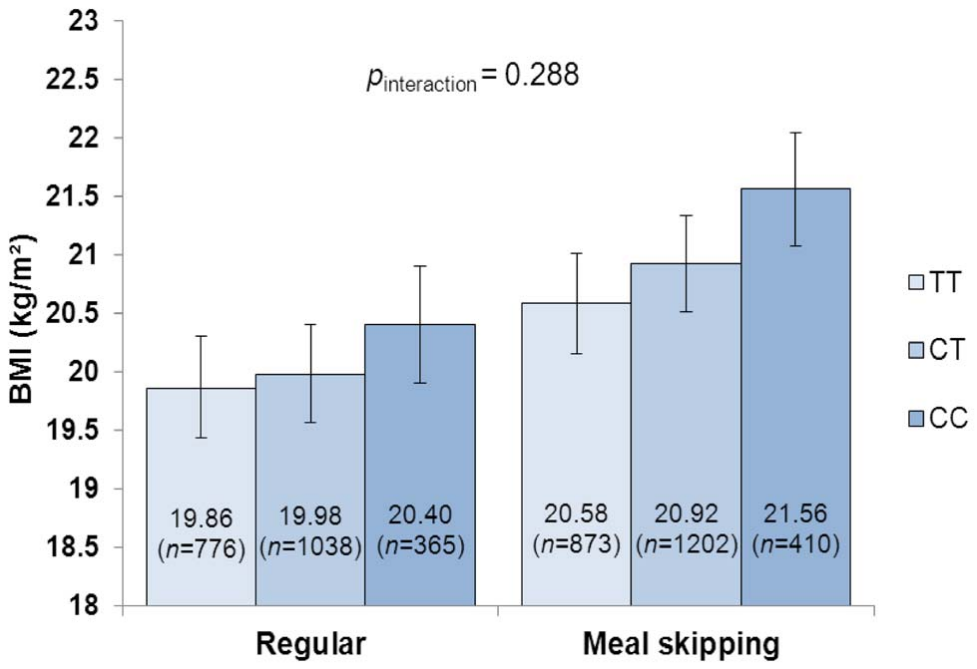
Interaction between *FTO* rs1421085 genotypes (additive model) and meal patterns on BMI. Mean BMI values with 95% confidence interval error bars are presented.

**Figure 4:**
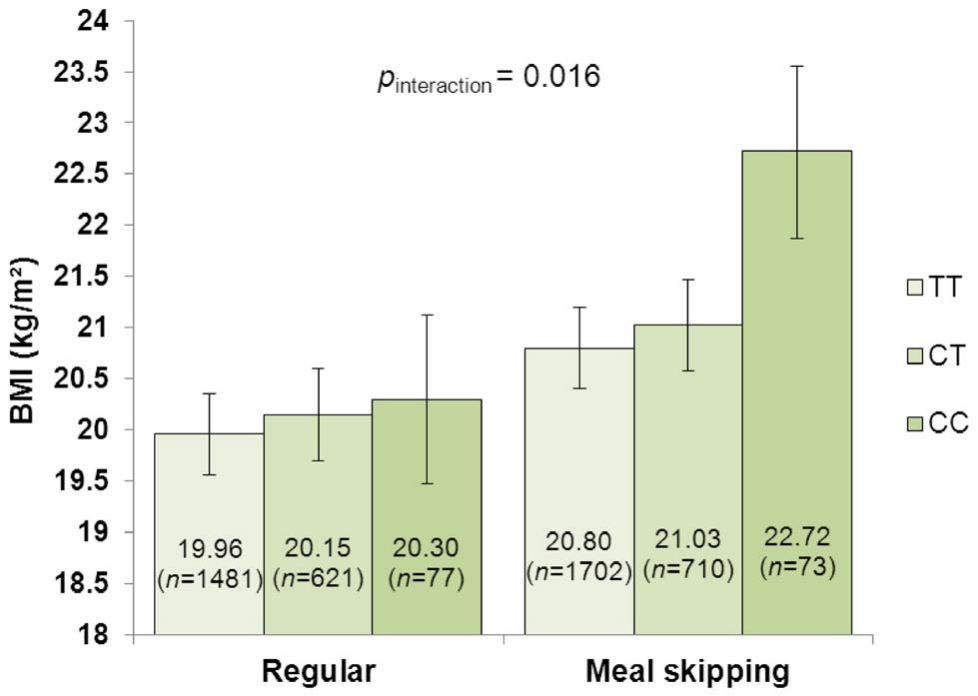
Interaction between *MC4R* rs17782313 genotypes (additive model) and meal patterns on BMI. Mean BMI values with 95% confidence interval error bars are presented.

We then examined interaction effects of meal frequencies and genotype groups (high- and low-risk groups based on GRS, and *FTO* rs1421085 and *MC4R* rs17782313 genotypes under an additive model) on the mean values of BMI using ANOVA adjusted for gender and pubertal stage. Among the meal skippers, the difference in BMI between the individuals with a high GRS (≥8 BMI-increasing alleles) and those with a low GRS (<8 BMI-increasing alleles) was 0.90 (95% CI 0.63, 1.17) kg/m^2^, whereas in the regular eaters, this difference was only 0.32 (95% CI 0.06, 0.57) kg/m^2^ (*p*_interaction_  = 0.003; Figure 2). Similarly, the difference in BMI between the individuals carrying two copies of the *FTO* rs1421085 risk alleles and those with no risk allele was pronounced in the meal skippers (0.98 kg/m^2^) compared with regular eaters (0.54 kg/m^2^) but the interaction was non-significant (*p*_interaction_  = 0.288; Figure 3). Per-allele effects of the *FTO* variant were 0.24 (95% CI 0.05, 0.42) kg/m^2^ for regular eaters and 0.46 (0.27, 0.66) kg/m^2^ for meal skippers. However, gender-stratified analysis showed that the interaction between the *FTO* rs1421085 and meal frequencies on BMI was significant in boys (*p*_interaction_  = 0.015) but not in girls (*p*_interaction_  = 0.617) (data not shown). Furthermore, the difference in BMI between the carriers of the two *MC4R* rs17782313 risk alleles and non-carriers was elevated to 1.92 kg/m^2^ in meal skippers, whereas in regular eaters, the difference was reduced to 0.34 kg/m^2^ (*p*_interaction_  = 0.016; Figure 4). Per-allele effects of the *MC4R* variant were 0.18 (95% CI -0.06, 0.41) kg/m^2^ for regular eaters and 0.47 (95% CI 0.22, 0.73) kg/m^2^ for meal skippers.

## Discussion

In the present study, we investigated the association of eight common SNPs with BMI and the possible modifying effect of meal frequency on genetic susceptibility to obesity in a genetically and culturally homogeneous population of 16-year-old Finnish adolescents. Besides using a genetic risk score, we separately analyzed effects of two well-established obesity loci, *FTO* and *MC4R*, on BMI. We showed that regular meal frequency attenuates genetic predisposition to increased BMI in terms of both single gene variants and a multiple-locus indicator. For the *FTO* rs1421085 variant, gender-specific interaction effects were observed.

As a whole, each additional BMI-increasing allele in the genetic risk score was associated with an increase in BMI of 0.21 kg/m^2^ which corresponds to a 0.61 kg increase in body weight for a person of 170 cm height. In adolescents who ate five meals a day, the BMI increase per risk allele was attenuated to 0.15 kg/m^2^ (0.43 kg), whereas among meal skippers this effect was increased to 0.27 kg/m^2^ (0.78 kg). A significant modifying effect of the regular meal frequency was observed also using the genetic risk score as a dichotomous variable and comparing high- and low-risk groups. Further, each risk allele at *FTO* rs1421085 increased BMI with 0.36 kg/m^2^ (1.04 kg) in adolescents overall, but this effect was diminished to 0.24 kg/m^2^ (0.78 kg) among regular eaters and elevated to 0.46 kg/m^2^ (1.33 kg) among meal skippers. The effect of each *MC4R* rs17782313 risk allele on BMI in the whole study population was 0.32 kg/m^2^ (0.92 kg); however, the BMI increase in meal skippers (0.47 kg/m^2^ [1.36 kg]) was nearly three-fold compared with the BMI increase in regular eaters (0.18 kg/m^2^ [0.52 kg]).

Recently, we showed the utility of environmental factors and low predictive value of common genetic variants in estimating the risk of child and adolescent obesity in newborns [22]. However, there are individuals with multiple genetic risk variants or rare mutations that seriously affect metabolic pathways leading to obesity. It is increasingly important to understand how environmental factors and lifestyle may modify the impact of genetic factors. Although the association between meal frequencies and obesity is relatively well-studied, this is, to our knowledge, the primary work considering the combined effect of meal frequencies and common genetic variants on body mass index in the field of gene-environment (gene-diet) interactions. In our analyses, meal frequencies were similarly distributed across genotypes and were, thus, independent. Nonetheless, food preferences and habitual dietary intakes seem to have both environmental and genetic foundations [23], [24]. In an ethnically diverse sample of overweight and obese adults with type 2 diabetes, risk alleles at *FTO* rs1421085 predicted more eating episodes a day [25]. In obese women showing extreme snacking behavior or use of excessive portion sizes, de Krom et al. [26] identified common allelic variations in the cholecystokinin gene, the leptin gene (*LEP*) and its receptor gene (*LEPR*) to be associated with abnormal eating habits. Moreover, Bienertová-Vasků et al. [27] found that common variations in the *LEP* and *LEPR* genes were associated with specific eating patterns, mainly in respect of timing of eating, independently of BMI. According to de Castro [28], genetic differences account for 44% of the variance in meal frequency. The suggested mechanistic explanations for the negative association between meal frequency and body weight have been related to the regulation of food intake, an increased thermogenic effect of food, and postprandial glucose and insulin responses [12].

In Western populations, the escalation of obesity has coincided with an increase in the prevalence of irregular meal patterns [13]. Nowadays, skipping meals is relatively common behavior in adolescence [29]. Breakfast is the meal most often skipped and girls are found to be more prone than boys to meal-skipping behavior [30], which was also seen in the NFBC1986 adolescents (the prevalence of meal skipping pattern 59.5% and 46.4%, respectively). The perceived meal-skipping of peers and family members, especially mothers, has been found to promote similar behavior among adolescents [31]. Besides being a risk factor for obesity, negative effects of meal skipping on adolescent wellbeing include poorer nutrient intake, compromised learning and academic performance and mental distress [32]–[34]. It is noteworthy that meal skipping might be used as a method for weight control [35] which complicates assessment of temporal relation between increased BMI and irregular meal frequencies. Since eating habits in adolescence seem to track into adulthood [36], nutrition interventions aimed at reducing meal skipping among adolescents are called for.

Our study has both strengths and limitations. First, the follow-up participation rates in the NFBC1986 were exceptionally high which reduces potential selection bias. Second, the anthropometrics were clinically measured which enhances the accuracy of the findings. With regard to limitations, the clinical examination was conducted at age 16 and participants were white; therefore, the results may not be applicable to other age or ethnic groups. The cross-sectional study design does not permit conclusions regarding causality. Since meal skipping has been found to be a popular dieting method among adolescents, especially in girls [34], the relationship between irregular meal frequencies and increased BMI may be partly due to reverse causation. On the other hand, for most adolescents unhealthy weight control behaviors are counterproductive and lead to weight gain over time [37]. A further limitation of the study is that the meal frequencies were assessed by a self-administered questionnaire with a limited choice of responses. As a result, there were no data on the composition of the daily meals and the actual number of daily snacks available for the analyses. The specific questions on eating habits were specially constructed for the NFBC data collection and were not validated against another dietary assessment method. However, since the inverse association between meal frequency and BMI is reasonably well established it can be interpreted as a qualitative support for the validity of meal frequency measurement [38].

## Conclusions

In the present study, we showed that meal frequencies can modify the effect of obesity-related genotype on BMI in adolescence. The results indicate that a regular five-meal-a-day pattern attenuates the effects of risk alleles on genetic susceptibility to increased BMI. In the light of current knowledge, promoting a regular eating pattern, i.e., five meals including breakfast, could be a potent obesity prevention strategy and bring forth other important health benefits.
